# Airborne microorganisms in muddy rain: microbe-mineral interactions and their ecosystem impact

**DOI:** 10.3389/fmicb.2026.1772201

**Published:** 2026-03-20

**Authors:** Azahara Navarro-Nieva, Fernando Martínez-Checa, Rafael Delgado, Jesús Párraga, M. Pilar Francino, Nuria Jiménez-Hernández, Ana del Moral

**Affiliations:** 1 Department of Soil Science, University of Granada, Granada, Spain; 2 Department of Microbiology, University of Granada, Granada, Spain; 3 Center for Biomedical Research (CIBM), Institute of Biotechnology, University of Granada, Granada, Spain; 4 Genomics and Health Area of FISABIO-Public Health Valencian Region Foundation for the Promotion of Health and Biomedical Research (FISABIO), Valencia, Spain

**Keywords:** African dust, atmosphere, ecosystem, extremophile, mineral-microbe interaction, red rain

## Abstract

The Sahara Desert and the Sahel region in North Africa contribute approximately 50–70% of global atmospheric dust emissions. Microorganisms can attach to dust particles and be dispersed into exogenous environments, being subsequently deposited by gravitational sedimentation (dry deposition) or through aqueous precipitation (wet deposition) also known as muddy rain. In the present work, five muddy rain samples were collected in Granada (Spain) during different episodes in 2021–2022. The SEM-EDX study demonstrated a high content of fine clay particles which may facilitate the atmospheric transport of microorganisms. The colonization of strategic microsites and the formation of mineral aggregates might be possible mineral-bacteria interactions. According to metagenomic analysis, *Pseudomonadota* (64%), *Bacteroidota* (13%), and *Bacillota* (6%) were the main phyla. At the genus level, extremophiles, plant-beneficial bacteria, and others involved in soil biogeochemical cycles have been described. Fourteen cultivable microorganisms were isolated and identified by means of 16S rRNA sequencing. Members of the phyla *Pseudomonadota, Bacillota, Actinomycetota* and *Bacteroidota* have been found. Among the isolates, *Stenotrophomonas rhizophila* and *Brevundimonas bullata* potentially exert beneficial effects at the ecosystem level. In general, muddy rain facilitates the transport and dispersal of microorganisms from different environments, with a potential positive influence on soils and vegetation in terrestrial ecosystems.

## 1 Introduction

Atmospheric dust is mainly generated by wind erosion due to soil deflation (Mahowald et al., 2005; Zobeck and Van Pelt, 2011). Arid, semi-arid, and desert regions act as global dust-source areas due to the lack of vegetation cover and frequent natural phenomena such as strong winds associated with dust storms. The Sahara Desert and the Sahel region in North Africa contribute approximately 50–70% of global atmospheric dust emissions (Luo et al., 2003; Middleton, 2017). In recent years, global warming linked to climate change has increased the frequency of dust storms that affect air quality and ecosystems (Zhou et al., 2023).

The atmosphere, due to its vast volume, constitutes the largest biome on planet Earth, although it remains almost unknown in terms of microbiology (Péguilhan et al., 2023). It represents one of the most inhospitable environments for life, owing to high levels of UV radiation, desiccation, extreme temperatures, and the presence of free radicals (Kellogg and Griffin, 2006). Microorganisms inhabiting this unique ecosystem may exhibit a high tolerance to harsh environmental conditions, being considered as stress-tolerant or extreme-condition-adapted (Aguilera et al., 2021).

Microorganisms can attach to suspended atmospheric dust particles and be dispersed into exogenous environments, thereby altering existing biodiversity in remote terrestrial and aquatic habitats (Barton et al., 2010). Their active role in organic matter decomposition processes and the transformation of chemical compounds essential for life is also relevant in soil biogeochemical cycles at the ecosystem level (Huddart and Stott, 2010).

The Iberian Peninsula is one of the main transport routes for atmospheric dust from the Sahara Desert and the semi-arid regions of the Sahel (Russo et al., 2020). Suspended dust is deposited onto terrestrial surfaces by gravitational sedimentation (dry deposition) or through aqueous precipitation (wet deposition) which can lead to muddy rain (formerly known as red rain or bloody rain) (Goudie and Middleton, 2006). The city of Granada (Spain), due to its proximity to North Africa, is frequently affected by both phenomena (Valenzuela et al., 2012).

Recently, microbial communities in muddy rain episodes have been described in a preliminary study in Granada (Navarro et al., 2024a). Likewise, the physical properties, mineralogy, and geochemical composition of atmospheric dust events recorded in the same region have also been described (Párraga et al., 2021; Molinero-García et al., 2022).

The aim of this work is to study the bacterial communities of muddy rain associated with dust events in the city of Granada (Spain) and to assess their possible influence at the ecosystem level. To date, the microbiology of muddy rain has been scarcely investigated (Itani and Smith, 2016; Navarro et al., 2024a). For this purpose, muddy rain samples were collected in Granada (Spain) during several episodes recorded in 2021 and 2022. This work is focused on the study of microbial communities and, to a lesser extent, the accompanying mineral particles (morphology and mineralogy), along with possible microbe-mineral interactions, which represents a novel approach. The use of multiple advanced techniques (16S rRNA gene sequencing, SEM-EDX microscopy...) has been essential for microbial characterization.

## 2 Materials and methods

### 2.1 Sampling site

**Figure 1:**
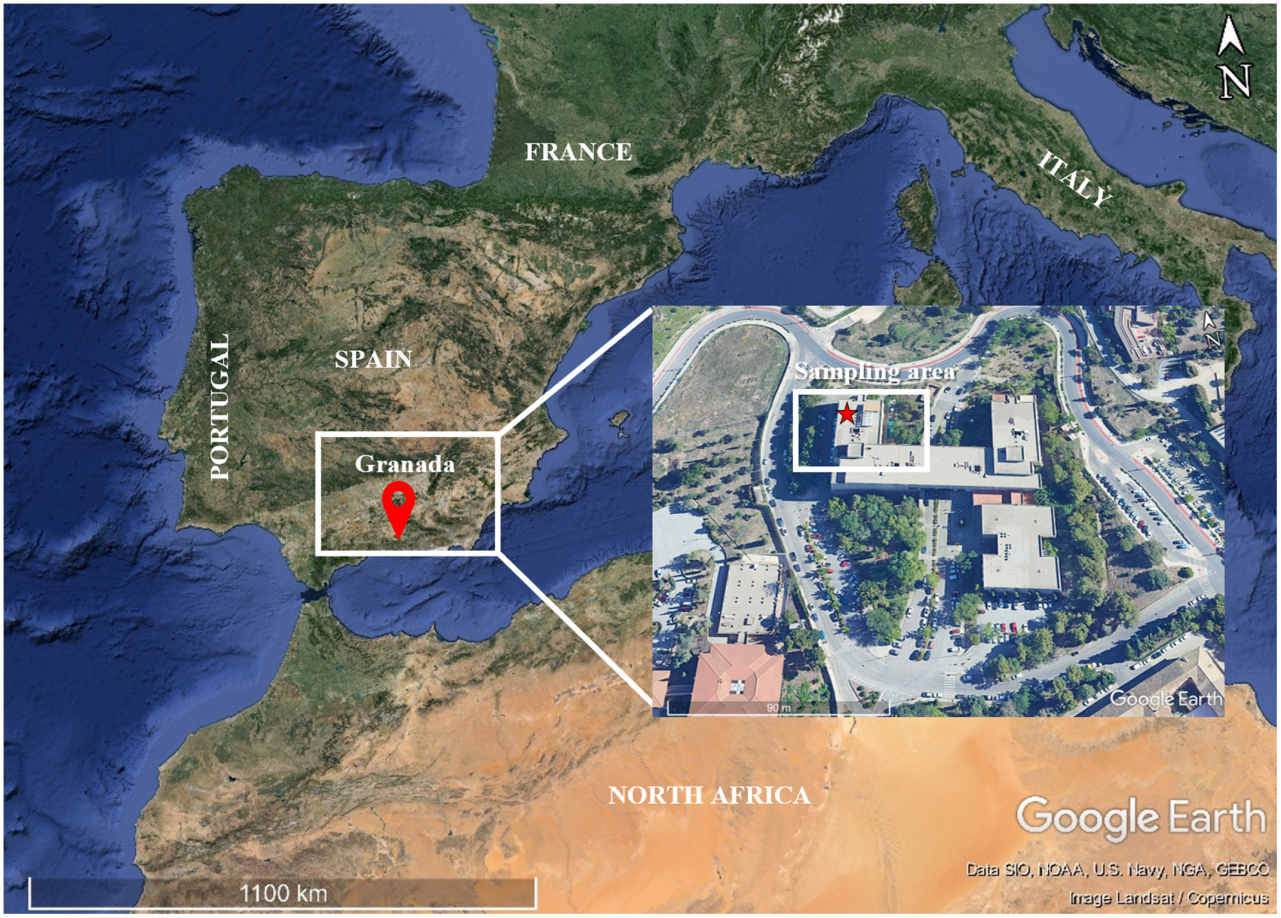
Location of the study area in Granada (southern Spain). Aerial view of the sampling area from the terrace of the Faculty of Pharmacy (University of Granada). Images retrieved from Google Earth.

The study area was the metropolitan region of the city of Granada (Spain), located in the southeast of the Iberian Peninsula, approximately 50 km from the Mediterranean Sea (37 ° 08′ 59″ N, 03 ° 37′ 59″ W) (Figure 1). The climate of the region is Mediterranean-continental and is influenced by its orography, as it is located on an intra-Betic depression and close to the Sierra Nevada mountain range. Annual rainfall is around 400 mm, and the mean annual temperature tends to reach values close to 16 °C (Agencia Estatal de Meteorología, 2025).

### 2.2 Identification and characterization of dust events associated with muddy rain episodes

**Figure 2:**
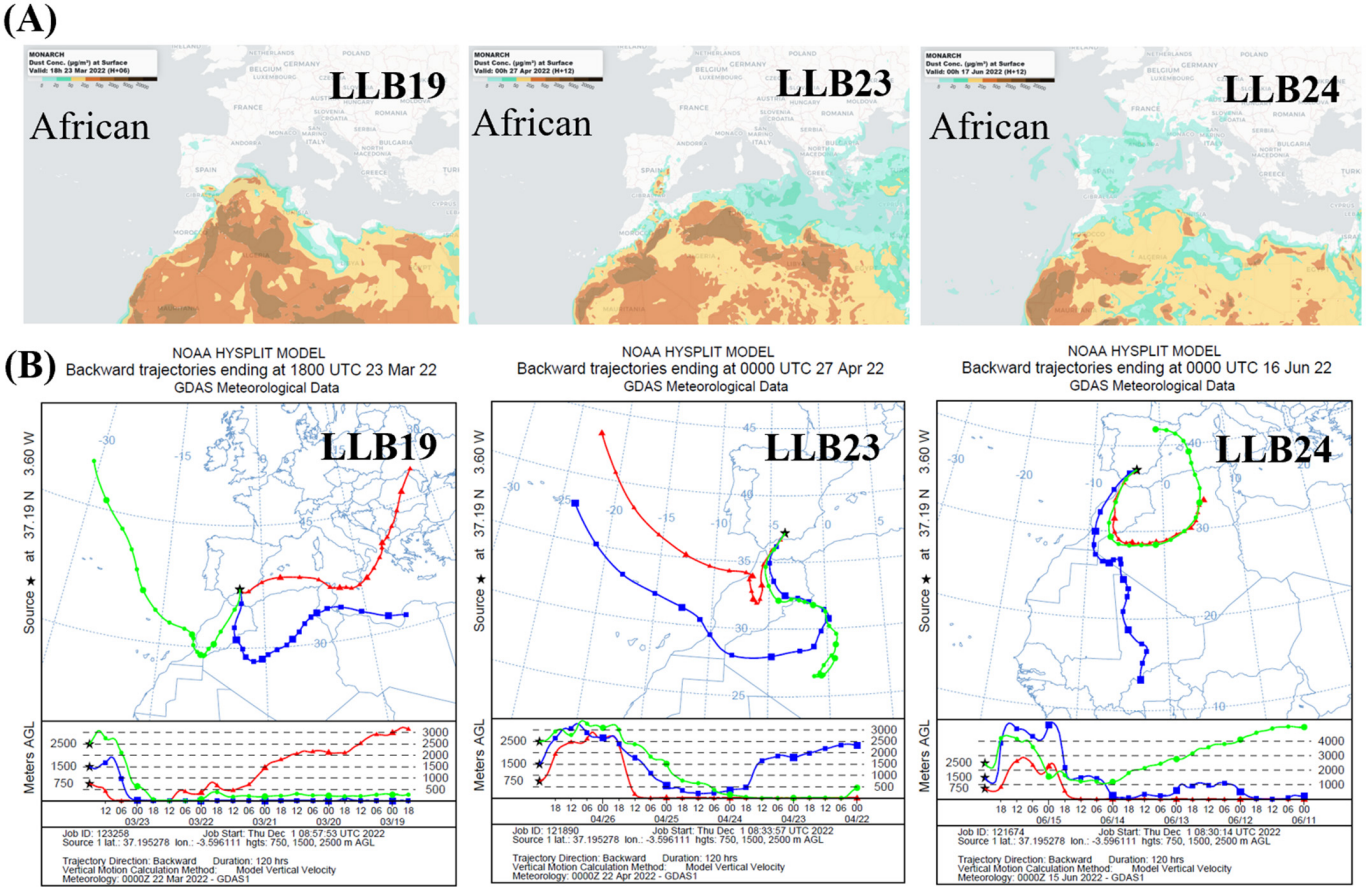
Identification of dust-source origin of three events associated with muddy rain samples LLB19, LLB23, and LLB24. **(A)** Dust concentration maps (MONARCH model, dust surface concentration μg/m3). **(B)** Backward trajectories: 750 m (red), 1,500 m (blue) and 2,500 m (green).

Dust events linked to each rain episode originated from Africa (Table 1), confirmed by official data from the southeast Iberian Peninsula (Ministerio para la Transición Ecológica y el Reto Demográfico, 2024). The provenance of air masses was determined based on backward-trajectory analysis using the HYSPLIT model (Stein et al., 2015; Rolph et al., 2017). It was calculated for 5 days using vertical velocity and altitudes of 750, 1,500, and 2,500 m above sea level. The backtracking distance of each dust event (km) was estimated using Google Earth. In addition, dust concentration maps were obtained with the MONARCH model (Figure 2) (Pérez et al., 2011; Klose et al., 2021).

**Table 1:** Description of muddy rain samples.

**Sample**	**Sampling Date**	**Date or period of the event**	Dust ^*^source	**Rain (mm)**	**DD rate^+^ (g/m^2^)**	PM_10_^**^ (μg/m^3^)	PM_10_^**^ (μg/m^3^)
LLB1	02 Nov 2021	29 Oct 2021	African	12.4	0.166	33.9	–
LLB16	16 Mar 2022	14–17 Mar 2022	African	2.6	16.373	550.3	143.6
LLB19	28 Mar 2022	20 Mar−1 Apr 2022	African	12.6	11.369	99.3	33.5
LLB23	29 April 2022	26–29 Apr 2022	African	–	–	20.8	8.4
LLB24	21 June 2022	12–21 June 2022	African	2.7	0.841	49.6	13.9

^+^ Dust deposition (DD) rate ^*^Ministerio para la Transición Ecológica y el Reto Demográfico (2024), and NOAA Air Resources Laboratory (2024), ^**^Junta de Andalucía (2021).

The particulate matter < 10 μm (PM_10_) and < 2.5 μm (PM_2.5_) concentration levels were recorded during sampling. These values were estimated using data from some monitoring stations belonging to “Red de Vigilancia y Control de la Calidad del Aire de Andalucía” (Junta de Andalucía, 2021): *Granada Congresos* (Granada; 37 °9′56“ N, 3 °6′ 00” W), *Granada Norte* (Granada; 37 ° 1′ 44“ N, 3 ° 6′ 51” W) and *Ciudad Deportiva* (Armilla; 37 ° 8′ 08“ N, 3 °7′ 09” W).

### 2.3 Collection of muddy rain

Sample collection was conducted on the rooftop terrace of the Faculty of Pharmacy (University of Granada), a 10-meter-high building situated in the metropolitan area of Granada city (37 °11′ 43″ N, 3 °35′ 46″ W; 760 m a.s.l.) (Figure 1). In total, five muddy rain samples (Table 1) were collected as wet deposition using a passive collector (precipitation), located on a two-meter mast support. Subsequently, rainwater was transferred into sterile 50 mL plastic containers after resuspension of the particulate matter by manual agitation. The collector consisted of a standard borosilicate glass vessel (17.5 cm in diameter and 9 cm in height), covered with a stainless-steel square mesh to prevent bird perching and nesting.

The passive collector was previously sterilized in an autoclave (121 °C, 20 min). Samples were obtained after muddy rain episodes recorded from November 2021 to June 2022. Additionally, a rainwater control (RC) sample was collected during a day without a dust event (20 Feb 2022) in order to characterize microorganisms transported in “clean” rainwater.

Muddy rain sampling (Table 1) was performed after the aqueous precipitation associated with each dust event had occurred—either during the dust intrusion or between 2 and 4 days later. Sampling duration varied depending on meteorological conditions (between 5 and 24 h).

The total volume of each rain episode was estimated from the amount of water collected in the passive collector. The dust deposition rate (DD) was calculated based on the surface area of the passive collector and the volume of water containing particulate matter, according to the procedure described by (Navarro Nieva 2025).

### 2.4 Electron microscopy analyses

The study was carried out by scanning electron microscopy (SEM) using the following equipment: (1) ESEM-FEI-Quanta equipped with an EDS-XFlash-Bruker detector, (2) FESEM-GEMINI with an EDX-OXFORD10 detector, and (3) SEM-TESCAN-AMBERX with an EDS-Oxford detector, all belonging to the Center for Scientific Instrumentation (CIC) of the University of Granada (CIC-UG, 2024). The chemical composition was determined by EDX microanalysis and mapping techniques, using mineral standards (CIC-UG, 2024) and other data sources for interpretation (Severin, 2004). To facilitate bacterial observation, samples were first treated with 2.5% glutaraldehyde in 0.1 M cacodylate buffer and 1% osmium tetroxide. Subsequently, they were filtered through a Millipore filter (0.2 μm pore size) to retain bacteria and mineral particles. The filter was dehydrated with ethanol, dried by the critical-point method, and finally coated with carbon (Kuo, 2014). Particle Feret diameter measurements for granulometric analysis were performed manually on the images using a ruler (>500 particles measured per sample).

### 2.5 Metagenomic analyses

DNA extraction and metagenomic sequencing were performed at the Genomics and Health Area of FISABIO (Valencia, Spain). Analyses were only performed on samples LLB1, LLB16, LLB19 and LLB24; the quantity required was not obtainable in sample LLB23.

#### 2.5.1 DNA extraction

Samples were centrifuged at 13,000 rpm for 5 min and the pellet was collected. Bacterial DNA from the pellet was extracted using the PureFood GMO Extraction and Authentication Kit on a Maxwell RSC robot (Promega).

#### 2.5.2 Sequencing of 16S rRNA gene amplicons

To obtain amplicons of the V3-V4 region of the 16S rRNA gene and prepare sequencing libraries, the Illumina protocol (16S Metagenomic Sequencing Library Preparation, Cod 15044223 RevA) was followed, with slight modifications due to the low initial amount of DNA (15 μl of amplicons and 12 amplification cycles were used in the index PCR that is performed to label the amplicons with dual adapters and indexes that allow for multiplex sequencing). The indexed amplicons sequencing was carried out on an Illumina MiSeq sequencer using the MiSeq Reagent kit v3 in a 2 x 300 cycle run.

Meta-taxonomic analysis of the sequences was performed with qiime2 (Bolyen et al., 2019). Noise reduction, paired-end joining, and chimera removal were performed with DADA2 (Callahan et al., 2016). The Silva138 database was used for taxonomic assignment (Quast et al., 2013).

#### 2.5.3 Indexes of bacterial diversity

Venn diagrams were obtained by using Venny v.2.1 tool (Oliveros, 2015). Alpha diversity comprised the Shannon (H') and the complementary Simpson (1-D) indexes. Beta diversity included the Jaccard (I_J_) and the Whittaker indexes (I_W_). Both alpha and beta diversity indexes were calculated with PAST v.4.16c software (Hammer et al., 2001).

### 2.6 . Study of cultivable microorganisms

Analyses were only performed on samples LLB1, LLB16, LLB19 and LLB24; the quantity required was not obtainable in sample LLB23.

#### 2.6.1 . Isolation of cultivable microorganisms from filters

Muddy rain samples were immediately processed after each precipitation episode. An aliquot (50 mL) of each sample was filtered through 0.2 μm (pore size) Millipore filter, after resuspending the particulate matter. Thereafter, each filter was deposited on a 10% Trypticase Soy Agar (TSA) medium and incubated at room temperature for several days. The serial dilution method (0.9% NaCl) was used for the isolation of microorganisms, and a preliminary identification was carried out by Gram staining. Strains were stored in Eppendorf tubes containing 0.75 mL of Trypticase Soy Broth (TSB) medium and 0.25 mL of glycerol (87%) at −80°C.

#### 2.6.2 . Identification of isolated cultivable microorganisms

The isolated microorganisms were identified by partial sequencing of the 16S rRNA gene. DNA extraction was performed using the Xtrem Biotech DNA extraction kit^1^, according to the procedure described by (Martín-Platero et al. 2007). Subsequently, universal bacterial primers F27 and R1492 were used for PCR amplification. The resulting PCR products were purified using the X-DNA purification kit (QIAquick PCR Purification Kit 250).

The 16S rRNA gene Sanger sequencing was carried out at STAB VIDA (Portugal). The obtained sequences were visualized using Chromas v.2.6.6 software (Technelysium, 2024) and compared to reference 16S rRNA gene sequences available in the GenBank, EMBL and DDBJ databases through the BLAST search and the EzBioCloud server (Johnson et al., 2008; Chalita et al., 2024).

## 3 Results

### 3.1 Identification of dust-sources and characterization of muddy rain episodes

Samples were collected during muddy rain episodes linked to five dust events with an African provenance (Table 1). Figure 2 shows dust concentration maps and backward trajectories for three samples.

The distance traveled by the air masses associated with each episode exhibited variability (Figure 2 and Table 2). The backward trajectory lengths ranged from approximately 2,300 to 12,700 km. The highest values generally corresponded to air masses at greater altitudes (2,500 m), whereas the lowest were related to air masses at minor altitudes (750 m).

**Table 2:** Distance of the backward trajectories (km) from dust events. data obtained from google earth.

**Sample**	**Height (m)**			
	**750**	**1,500**	**2,500**	**Mean**
LLB1	2,376	2,739	12,697	5,937
LLB16	3,997	3,359	4,980	4,112
LLB19	3,823	3,434	4,158	3,805
LLB23	3,021	3,922	3,129	3,357
LLB24	2,431	2,972	3,871	3,091

Muddy rain episodes occurred throughout the seasons, and precipitation was variable (Table 1). Samples were collected in autumn (one), spring (three), and summer (one). The amount of precipitation collected during each episode ranged from 2.6 to 12.6 mm.

Dust deposition rates (DD) and particulate matter concentration (PM_10_ and PM_2.5_) were also variable (Table 1). Samples LLB16 and LLB19 exhibited the highest deposition rates (≈10–15 g/m^2^), whereas the others showed considerably lower ones (< 1 g/m^2^). Similarly, the highest values of particulate matter concentration were also found in sample LLB16 (PM_10_ = 550.3, PM_2.5_ = 143.6), while the lowest corresponded to sample LLB23 (PM_10_ = 20.8, PM_2.5_ = 8.4).

### 3.2 Electron microscopy analyses

**Figure 3:**
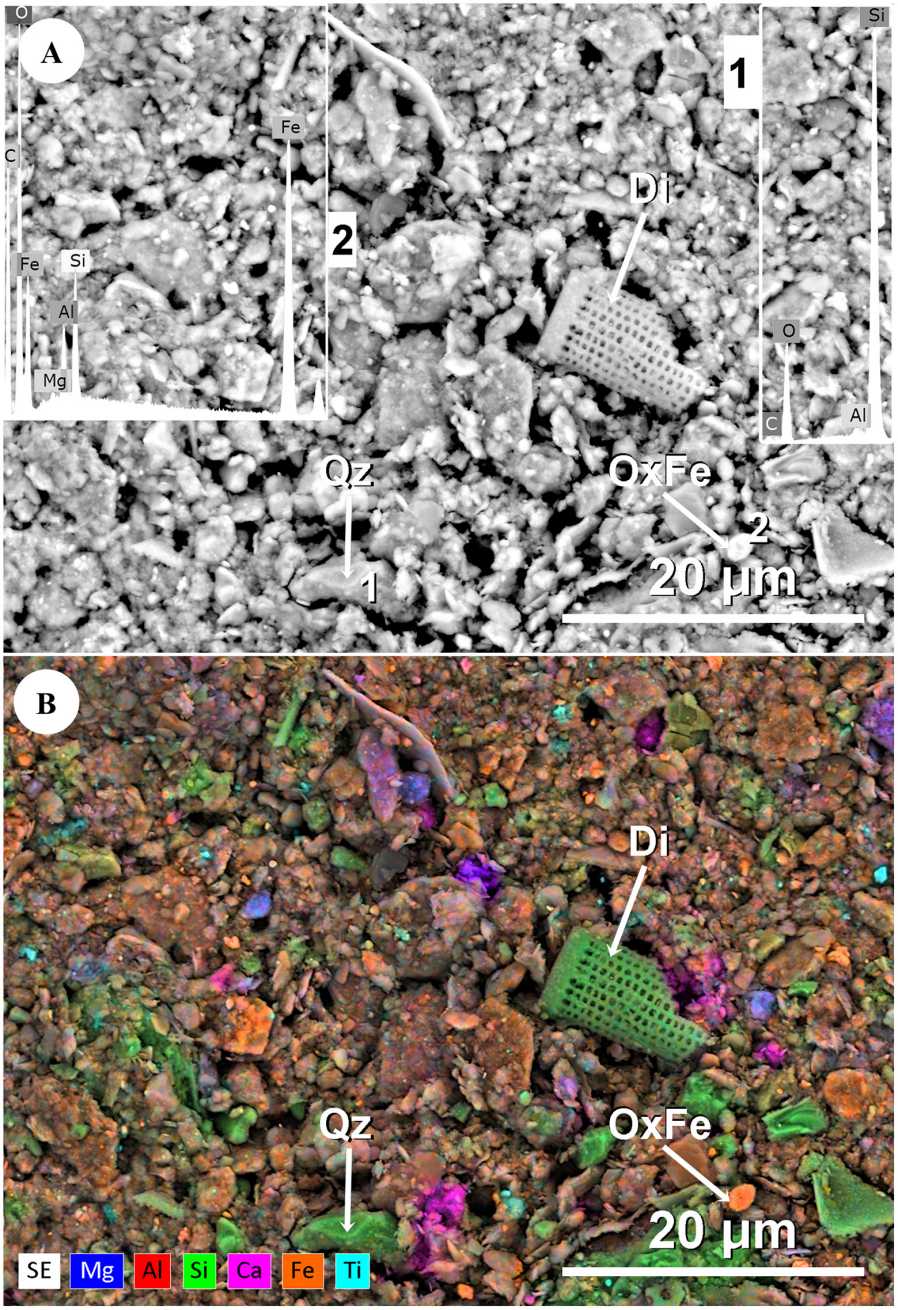
Sample LLB24. **(A)** Backscattered electron signal (BSE). Quartz grain (1) (Qz) of around 8 μm with a pseudoprismatic morphology and a detrital appearance, in a field of clay (< 2 μm) and fine silt particles (2–20 μm); EDX1: Si, O, and (Al). Particle rich in iron oxyhydroxide (2) (OxFe) of around 2 μm with an oval morphology; EDX2: Fe, Si, Al, O, and (Mg). Diatom valve (Di) fragment of around 12 μm with a centric morphology. Equipment: ESEM-FEI Quanta, EDS-XFlash Bruker. **(B)** Secondary electron signal (SE). Same field of view (Figure 3A), shown in false color. Chemical element map: Mg, Al, Si, Ca, Fe, and Ti. Equipment: ESEM-FEI Quanta, EDS-XFlash Bruker.

Muddy rain samples were granulometrically composed of approximately 90% clay (particles < 2 μm) and 10% silt (2–50 μm), with no sand content (> 50 μm). Figure 3A depicts a field of heterometric particles, predominantly fine (< 0.2 μm) and coarse (0.2–2 μm) clay, along with fine silt particles (2–20 μm) in minor proportion. The particles exhibited diverse morphologies, including laminar, pseudopolyhedral, pseudo-spherical, pseudo-ellipsoidal and fibrous shapes. In some cases, characteristic features such as rounded edges, subrounded surfaces, and mechanical marks were observed, denoting wind transport and aeolian modeling from the source area. Additionally, a fragmented diatom valve (Di) of around 12 μm with centric morphology was also recognized.

According to the chemical element map (Figure 3B), mineral particulate matter contained in muddy rain samples was mainly composed of Si, Al, and Mg; other elements such as Fe, Ca, and Ti were also detected. Mineralogical composition—inferred from the chemical element map and EDX microanalysis—would imply a high proportion of phyllosilicates and, to a lesser extent, quartz. Others such as carbonates, iron oxyhydroxides, and rutile might also be present. The bright appearance of particles in the backscattered electron signal (BSE) (Figure 3A) would also reflect a high atomic weight, justifying the presence of certain minerals such as iron oxyhydroxides or rutile, as well as surface coating on other particles with iron-rich phases.

**Figure 4:**
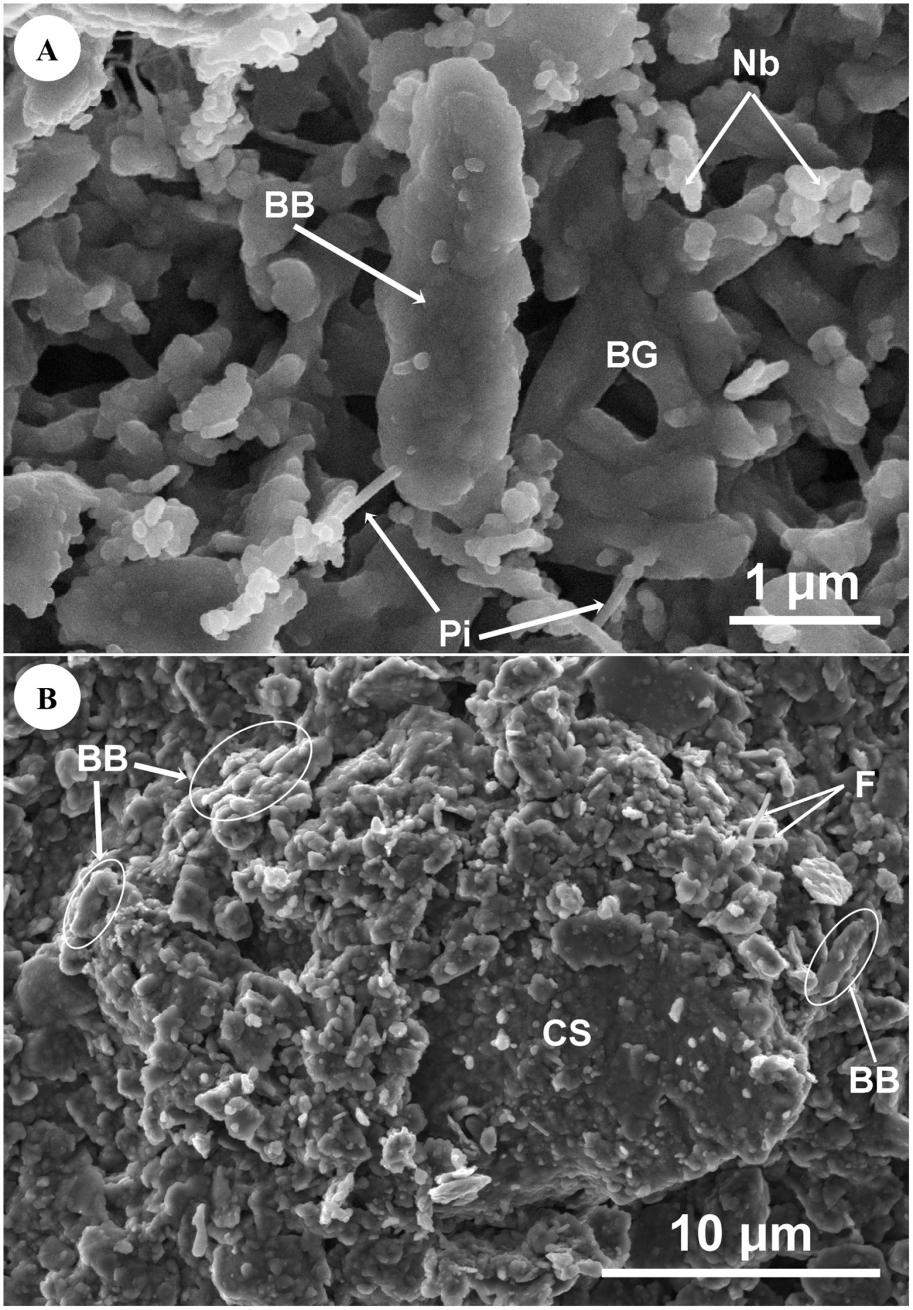
Samples LLB23 and LLB24. **(A)** Secondary electron signal (SE). Bacterial cell (BB) of approximately 3 μm, with possible pili (Pi), coated with fine clay mineral particles (< 0.2 μm). Frequent nanometer-sized bacterial-like structures (Nb) of around 80–200 nm are observed. The supporting filter (BG) is visible on the right quadrant. Equipment: FESEM-GEMINI. **(B)** Secondary electron signal (ETD). Particle of coarse silt (CS) coated with fine (< 0.2 μm) and coarse (0.2–2 μm) clay mineral particles. Bacterial cells (BB) of approximately 3 μm and possible filaments (F) can be identified within the ellipses. Equipment: ESEM-FEI Quanta.

Muddy rain can harbor microorganisms that interact with mineral particles, as demonstrated by SEM images. Figure 4A shows a bacterial cell (BB) of approximately 3 μm coated with fine clay, which is attached to other mineral particles potentially via *pili* (Pi). In Figures 4B, A coarse silt particle (CS) coated with both fine and coarse clay is observed. Possible filaments (F) as well as bacterial cells (BB) of around 3 μm coated with fine clay are also visible.

**Figure 5:**
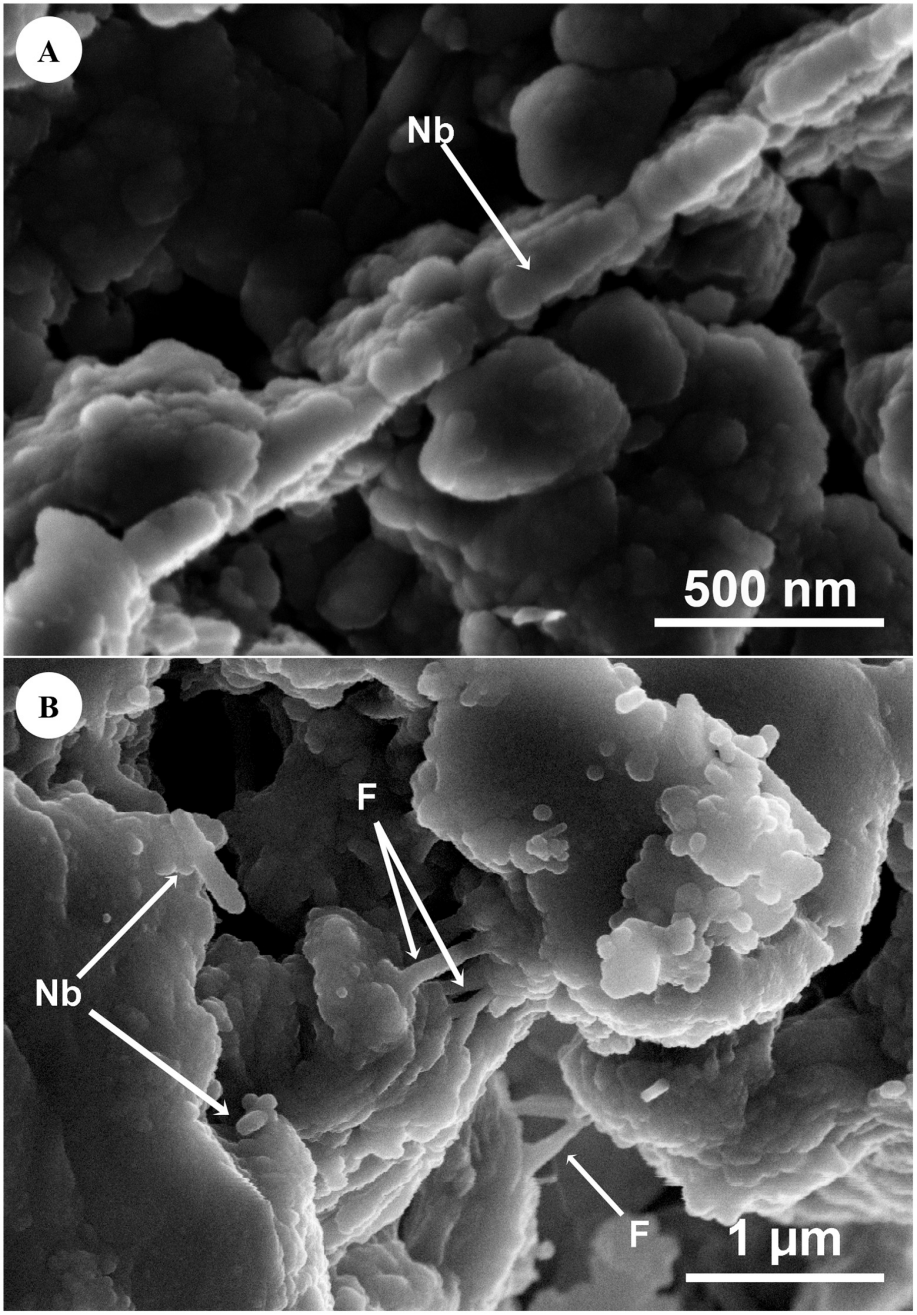
Sample LLB23. **(A)** Secondary electron signal (Axial). Nanometer-sized bacterial-like structures (Nb) of approximately 330 nm (mean diameter) showing a rod-shaped morphology, within a field of clay mineral particles (< 2 μm). Equipment: SEM-TESCAN AMBERX. **(B)** Secondary electron signal (SE). Nanometer-sized bacterial-like structures (Nb) of around 80–350 nm with a rod-shaped morphology, occurring on microaggregates of mineral particles. Possible filaments **(F)** can be observed as bridging structures between mineral clusters. Equipment: FESEM-GEMINI.

The presence of nanometer-sized bacterial-like structures -which might be possible nanobacteria-in muddy rain was also confirmed by means of SEM. Figure 5A depicts a group of nanometer-sized bacterial-like structures (Nb) with an average size of ≈ 330 nm on mineral particles. In Figure 5B, nanometer-sized bacterial-like structures (Nb) of around 80–350 nm are also observed on a microaggregate of mineral particles, with possible filaments (F) acting as bridges between the mineral clusters.

### 3.3 Metagenomic analysis

**Figure 6:**
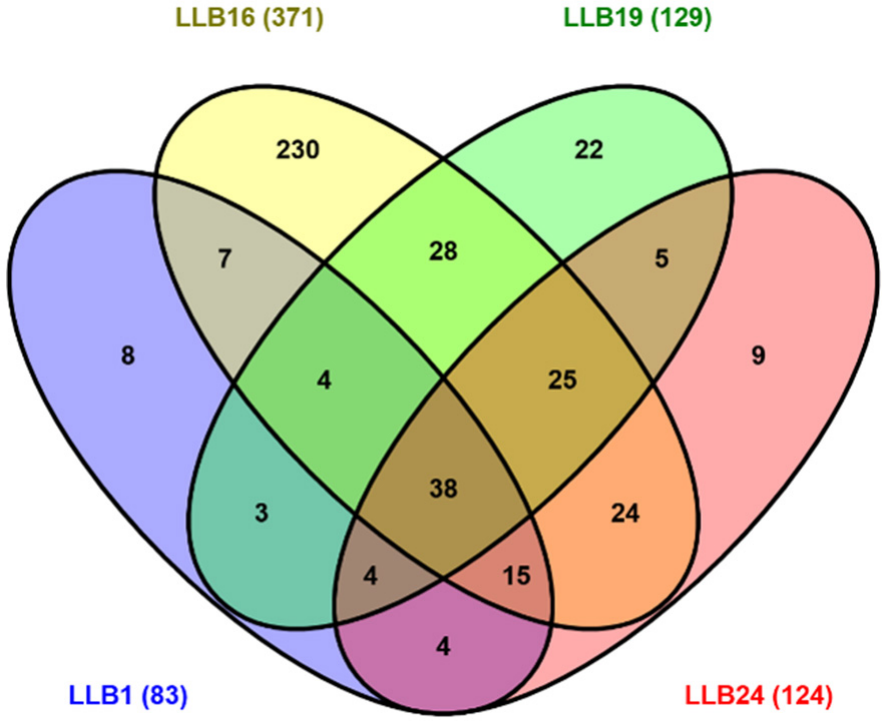
Venn diagram. Number of shared and specific genera in muddy rain.

The bacterial community was composed of 29 phyla and 563 genera (Table S1). The Venn diagram showed that only 38 genera were shared among all samples (Figure 6). In contrast, the number of specific genera was considerably higher in sample LLB16 (230), whereas it remained much lower and relatively similar in the rest (from 8 to 22).

**Figure 7:**
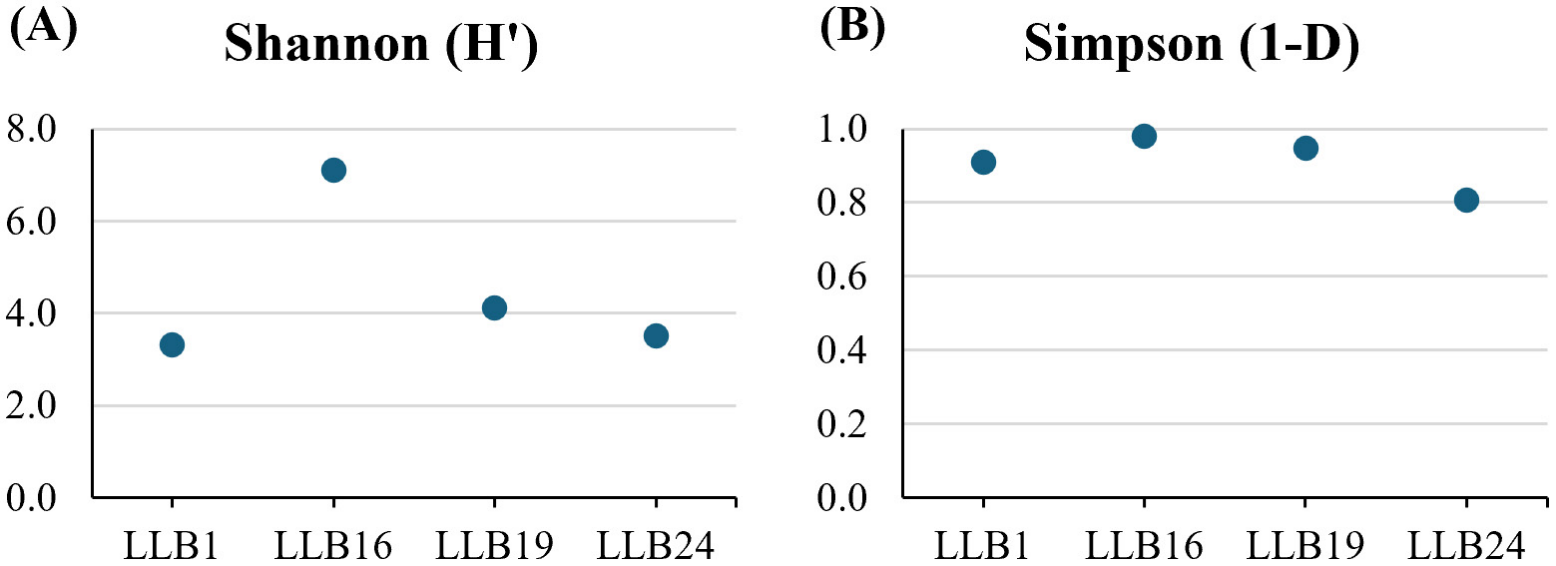
Indexes of alpha diversity of the bacterial community in muddy rain: **(A)** Shannon (H') and **(B)** Simpson (1-D).

The alpha diversity analysis revealed differences among the samples (Figure 7). The Shannon index (H') varied from 7.11 (LLB16) to 3.32 (LLB1), indicating high diversity (H'>3) across samples. Similarly, the complementary Simpson index (1-D) yielded values close to 1 in all cases, showing high diversity too. The highest value corresponded to sample LLB16 (0.98), whereas the minimum was observed in sample LLB1 (0.8).

The beta diversity analysis demonstrated pronounced heterogeneity among the bacterial communities across samples (Table 3). Based on the Jaccard index, the highest similarity was observed in the pair of samples LLB1–LLB24 (0.41) and the lowest in the pair LLB1–LLB16 (0.15). Following the Whittaker index, the greatest dissimilarity occurred in the pair LLB1–LLB16 (0.74), while the lowest was found in the pair LLB1–LLB24 (0.42).

**Table 3:** Indexes of beta diversity of the bacterial community in muddy rain.

	**LLB1**	**LLB16**	**LLB19**	**LLB24**
(a) Jaccard Index (I_J_)				
LLB1	1.000	0.149	0.306	0.405
LLB16		1.000	0.224	0.229
LLB19			1.000	0.380
LLB24				1.000
(b) Whittaker Index (I_W_)				
LLB1	0.000	0.740	0.531	0.424
LLB16		0.000	0.634	0.627
LLB19			0.000	0.449
LLB24				0.000

**Figure 8:**
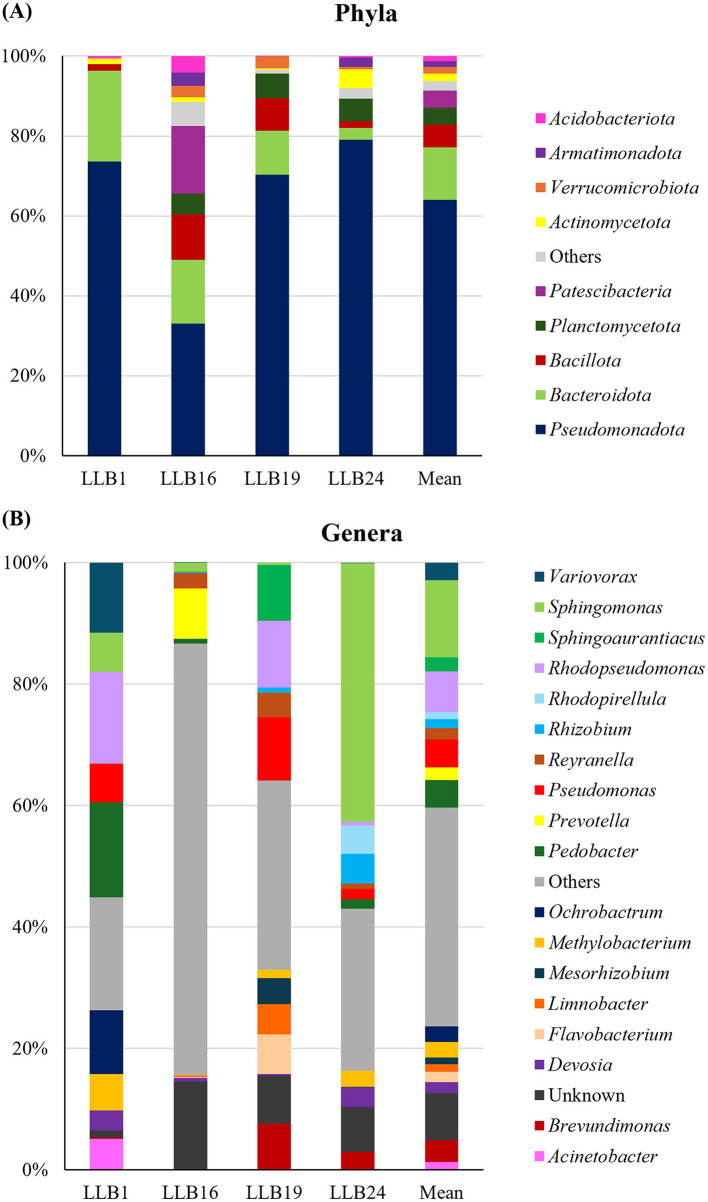
Study of the bacterial community in muddy rain. **(A)** Phyla and **(B)** genera with an average relative abundance > 1%.

According to the mean relative abundance, the dominant phyla were *Pseudomonadota* (64%), *Bacteroidota* (13%), and *Bacillota* (6%) (Figure 8A). At the genus level, *Sphingomonas* was the most abundant (13%), followed by *Rhodopseudomonas* (8%) and *Pseudomonas* (7%) (Figure 8B).

### 3.4 Identification of isolated cultivable microorganisms

**Figure 9:**
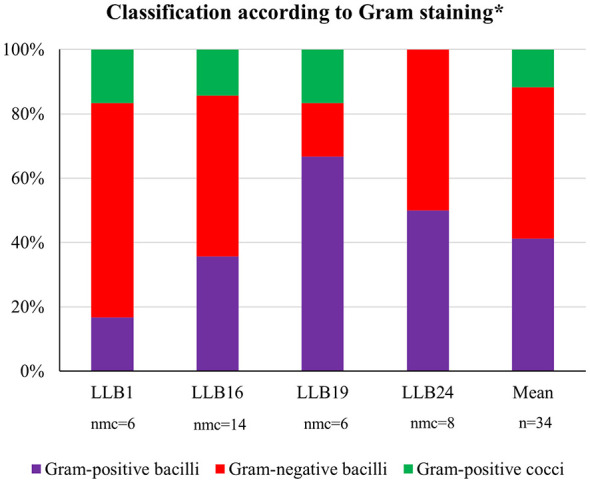
Isolated cultivable microorganisms. Classification according to Gram staining (* Number of cultivable microorganisms (nmc) isolated per sample).

The number of isolated cultivable microorganisms (nmc) from muddy rain samples was thirty-four (Figure 9). Most of them were bacilli (88%), including Gram-positive (47%) and Gram-negative (41%) strains; the remaining were Gram-positive cocci (12%). Besides, around 45% of the Gram-positive bacilli were spore-forming.

In total, fourteen cultivable microorganisms were taxonomically identified (Table 4). Additionally, the microorganism isolated from the rainwater control (RC) sample was also identified.

**Table 4:** Identification of isolated bacteria in muddy rain. The taxa, strains and percentage identities of the 16S rRNA gene sequences are indicated.

**Sample**	**Taxon**	**Strain**	**Identity (%)**
RC	*Priestia endophytica*	2DT	100.00
LLB1	*Pseudomonas shirazensis*	SWRI56	100.00
LLB16	*Pseudomonas farris*	SWRI79	99.78
	*Acidovorax radices*	N35	100.00
	*Pseudomonas poae*	DSM 14936	99.56
	*Arthrobacter frigidicola*	MDT2–14	99.85
	*Flavobacterium caeni*	LM5	95.94
	*Pseudomonas mucoides*	P154a	99.78
LLB19	*Peribacillus frigoritolerans*	DSM 8801	99.64
	*Bacillus siamensis*	KCTC 13613	100.00
	*Peribacillus muralis*	DSM 16288	100.00
LLB24	*Massilia niabensis*	5420S−26	99.33
	*Stenotrophomonas rhizophila*	DSM 14405	100.00
	*Brevundimonas bullata*	IAM 13153	100.00
	*Brevundimonas subvibrioides*	ATCC 15264	100.00

The identified microorganisms mainly belonged to the phyla *Pseudomonadota (63%)* and *Bacillota* (31%), which were also predominant in the metagenomic analysis (Figure 8A). Furthermore, other minor phyla such as *Actinomycetota* and *Bacteroidota* (both 7%) were also detected.

**Figure 10:**
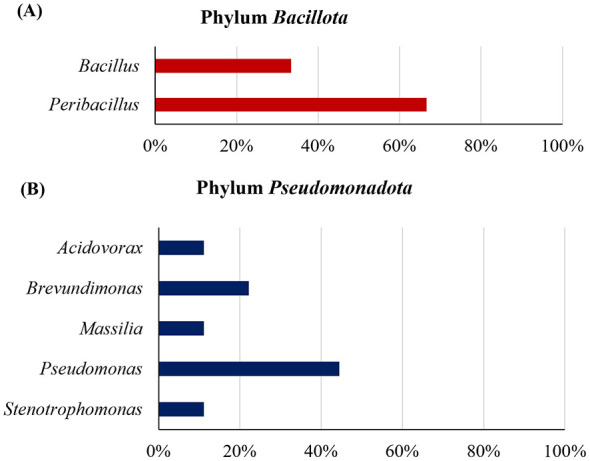
Isolated microorganisms (genera) belonging to the phylum: **(A)** *Bacillota* and **(B)** *Pseudomonadota*.

The genus *Pseudomonas* was the most abundant within the phylum *Pseudomonadota* (Figure 10), followed by *Brevundimonas, Stenotrophomonas, Acidovorax*, and *Massilia* in lower proportions. On the other hand, the genera *Bacillus* and *Peribacillus* were found within the phylum *Bacillota* (Figure 10), while *Arthrobacter* and *Flavobacterium* were the only representatives of *Actinomycetota* and *Bacteroidota*, respectively. All identified genera had already been detected in the metagenomic analysis (Supplementary Table 1 and Figure 8B).

## 4 Discussion

### 4.1 Identification of dust-sources and characterization of muddy rain episodes

Muddy rain episodes corresponded to dust events originating from North Africa (Table 1). According to backward trajectories, African air masses may also converge with others with Mediterranean, Atlantic, and/or regional provenance (Figure 2). The distance traveled by these air masses was variable and showed a decreasing trend throughout the study period (Figure 2; Table 2), potentially related to the seasonality of the episodes (Table 1).

The precipitation volume ranged from 2.6 to 12.6 mm, with similar values recorded for the pairs of samples LLB16–LLB24 (≈ 2.5 mm) and LLB1–LLB19 (≈ 12.5 mm) (Table 1). Previous studies reported that muddy rain episodes mainly occur in spring and autumn in the Iberian Peninsula, coinciding with the seasons of highest rainfall (Ávila et al., 1997; 2007; Castillo et al., 2017). In our case, most events were indeed recorded during spring, although precipitation volume was not directly related to seasonality. Interestingly, no relationship was either observed between precipitation volume and microbial composition. According to literature, rainwater plays a crucial role in preserving cellular integrity and supporting microbial replication under desiccation as well as other environmental stress conditions (Hu et al., 2018; Hallsworth, 2022). Thus, a higher rainwater volume would be expected to favor microbial communities although no evidence was detected.

The deposition rate of particulate matter in muddy rain samples ranged from < 1 to 16.4 g/m^2^ (Table 1). These values showed a positive correlation with PM_10_ and PM_2.5_ concentration and were consistent with previous studies of muddy rain episodes recorded in the Iberian Peninsula (Ávila et al., 1997, 2007; Fiol et al., 2005). As stated in the literature, higher deposition rates registered during African dust events contribute to enhance the transport and dispersal of microorganisms (Griffin et al., 2007). Our results would support this observation based on the positive relationship between the deposition rate and the microbial community as will be discussed later (Table 3; Figures 6, 7).

The PM_10_ concentrations ranged from ≈ 20 to 550 μg/m3 (Table 1). Samples LLB16 and LLB19 recorded values around 550 μg/m3 and 100 μg/m3, posing a threat to human health according to RD 34/2023 (Boletín Oficial del Estado, 2023). Similarly, PM_2.5_ concentrations varied from < 10 to ≈ 150 μg/m3 and the highest value also corresponded to LLB16 (Table 1). According to previous studies, particle size distribution during dust events may strongly influence airborne microbial populations (Polymenakou et al., 2008; Hara and Zhang, 2012; Stern et al., 2021). In our case, a direct relationship between particulate matter concentration and bacterial communities was also observed, as will be discussed hereinafter (Table 3; Figures 6 y 7).

### 4.2 Electron microscopy analyses

Muddy rain samples contained heterometric mineral particles with variable morphology. They were rich in clay particles (< 2 μm) and also contained silt (2–50 μm) (Figure 3), in proportions comparable to previous studies on muddy rain (Navarro et al., 2024a). Clay mineral particles possess a reduced size (< 2 μm) and a high specific surface area, providing numerous microsites and attachment points (Fomina and Skorochod, 2020) that may facilitate the atmospheric transport of microbes during dust events (Stern et al., 2021; Gat et al., 2022; Zhao et al., 2023; Lang-Yona et al., 2025).

Mineralogical composition -inferred from SEM morphology and chemical analysis- revealed dominance of phyllosilicates, quartz, and less abundant minerals such as carbonates, iron oxyhydroxides, and rutile (Figure 3). Phyllosilicates contain elements such as Na, Ca, and K, which may serve as nutrients for airborne microorganisms. Additionally, some mineral phases such as smectite, commonly found in muddy rain (Ávila et al., 1997) and African dust (Scheuvens et al., 2013), can retain water in their pores and interlayer spaces of the crystalline structure, thus creating potential microenvironments which might also favor microbial growth (Cuadros, 2017; Lang-Yona et al., 2025). On the other hand, the deposition of these minerals through rainfall may also supply nutrients influencing soil biogeochemical cycles and vegetation in terrestrial ecosystems (Ávila and Peñuelas, 1999), potentially affecting the soil microbiome.

Muddy rain samples also carried biological material, capable of dispersing into allochthonous ecosystems. Figure 3 shows a fragmented diatom valve (Di) with a centric morphology, possibly belonging to the genus *Aulacoseira*. This genus is a fresh-water taxon abundant in diatomite deposits from the Bodélé Depression, often used as an indicator of African dust sources (Chou et al., 2008; Scheuvens and Kandler, 2014). Its silica-rich composition might be attributed to biogenic opal (Figure 3B). According to literature, diatom valves can modify the mechanical and physicochemical properties of soils, thereby affecting the soil microbiome (Shiwakoti et al., 2002; Rodríguez and González-Rodríguez, 2013; Caicedo et al., 2019).

Interactions between microorganisms and mineral particles were also observed. Figure 4 suggests that bacteria (BB) may travel in air either adhered to particle surfaces via *pili* (Pi) or embedded in mineral aggregates. Such interactions have been previously reported in literature as passive or active transport mechanisms, enabling bacteria to potentially colonize microsites with better humidity and/or nutrient conditions (Halverson, 2005). On the other hand, bacteria may also promote aggregate formation via filamentous structures and/or biofilms (Maki et al., 2019; Párraga et al., 2021; Hu et al., 2023; Lang-Yona et al., 2025). Furthermore, these features might enhance particle stability and act as protective niches against atmospheric stressors such as UV radiation, high temperature, or desiccation (Amato et al., 2017; Maki et al., 2025).

Airborne microbes found in muddy rain may also possess adaptive mechanisms to environmental stress. Figure 5 depicts different examples of possible nanobacteria (Nb) (80–350 μm) attached to mineral particles. Nanobacteria are nano-sized microorganisms (50–500 nm) which may exhibit coccoid or bacillary morphologies similar to bacteria (Martel et al., 2014). These microbes were initially described as stress or resting forms of larger bacteria (Morita, 1988) and are also known as calcifying nanoparticles due to their biomineralization properties (Kajander et al., 2003). Nanobacteria have been previously identified in soils (Vainshtein and Kudryashova, 2000), the stratosphere (Sommer et al., 2004) and even meteorites (Benzerara et al., 2003). Additionally, they have recently been detected for the first time in muddy rain (Navarro et al., 2024a; Navarro Nieva et al., 2024).

### 4.3 Metagenomic analysis

Richness of bacterial genera revealed differences across muddy rain samples (Figure 6; Supplementary Table 1). The highest number of genera corresponded to LLB16 (371), considerably decreasing in the other samples (83–129). As mentioned earlier, particulate matter concentration may have an influence on the microbial community. In our case, the number of genera was strongly related to deposition rate as well as PM_10_ and PM_2.5_ concentration (Figure 6; Table 1).

Alpha diversity indexes differed in each episode (Figure 7). The greatest values were observed in LLB16, with high bacterial diversity as mentioned before. Interestingly, the higher diversity of bacterial genera was strongly related again to the greater deposition rates and particulate matter concentration (PM_10_ and PM_2.5_) (Figure 6; Table 1). This observation would also be valid for sample LLB19, with high alpha diversity values too.

Beta diversity indexes confirmed that bacterial communities were highly heterogeneous (Table 3). Deposition rate and PM_10_ concentration indicated again a clear relationship with similarity and/or dissimilarity among samples (Table 1). For example, the pair LLB1–LLB24 (0.41), showing the greatest similarity, exhibited lesser although similar deposition rates and PM_10_ concentration. In contrast, the pair LLB1–LLB16 (0.15), which had the lowest similarity, corresponded to episodes with highly contrasting deposition rates (< 1 and 16 g/m^2^) and PM_10_ concentrations (33.9 and 550.3 μg/m3). Thus, our results reaffirm that the community heterogeneity in muddy rain may depend again on both deposition rate and particulate matter concentration.

At the phylum level, *Pseudomonadota* (64%), *Bacteroidota* (13%), and *Bacillota* (6%) were dominant in muddy rain samples (Figure 8A). According to literature, the phylum *Pseudomonadota* was also the most abundant in almost all studies on muddy rain (Itani and Smith, 2016; Navarro et al., 2024a) and atmospheric dust (Sánchez De La Campa et al., 2013; Federici et al., 2018; Triadó-Margarit et al., 2019; González-Toril et al., 2020; Stern et al., 2021; Das et al., 2023; Hu et al., 2023). In contrast, *Bacillota* was only found to be predominant in several studies on atmospheric dust (Polymenakou et al., 2008; Sorkheh et al., 2022).

Microorganisms detected in muddy rain originated from different environments. Among the identified genera (Figure 8B), *Sphingomonas* was frequently found in desert air masses (Kellogg and Griffin, 2006; Federici et al., 2018; Petroselli et al., 2021; Das et al., 2023; Hu et al., 2023) while *Pedobacter, Pseudomonas*, and *Flavobacterium* were common in soils (Bernardet and Bowman, 2015; Maier, 2015; Margesin and Shivaji, 2015). Other genera such as *Limnobacter, Rhodopirellula, Rhodopseudomonas* and *Sphingoauranticus* were closely related to water bodies (Maier, 2015; Chen et al., 2016; Storesund et al., 2018; Jiang et al., 2019; Zhou et al., 2025).

Muddy rain can also harbor microorganisms able to cope with the harsh conditions in the atmosphere. Several taxa such as *Sphingomonas, Devosia, Mesorhizobium*, and *Methylobacterium* may include extremophiles and be resistant to different environmental stress conditions: high temperature, UV radiation, pH, desiccation and/or salinity (Halo et al., 2015; Dludlu et al., 2018; Asaf et al., 2020; Burcham et al., 2021; Zhang et al., 2021). Spore formation also constitutes a key resilience mechanism against environmental stress (Hu et al., 2023), typical of some genera such as *Bacillus* and *Peribacillus* found in our study (Table 4 and Supplementary Table 1).

Airborne microbes may also have special capabilities to ensure their survival during atmospheric transport. *Flavobacterium* and *Limnobacter* may include species with motility structures, facilitating the movement to potential microsites presumably with higher contents of moisture and nutrients (Bernardet and Bowman, 2015; Chen et al., 2016). In our case, the presence of bacteria with biological structures such as *pili*, clearly involved in transport and surface attachment, was also proven by SEM-EDX (Figure 4A). Additionally, other genera found in our study such as *Bacillus, Pseudomonas*, and *Rhizobium* (Table 4 and Supplementary Table 1) are known to produce extracellular polymeric substances (EPS) and biofilms, enhancing mineral aggregation and creating nutrient-rich microhabitats (Halverson, 2005; Gat et al., 2022; Netrusov et al., 2023; Lang-Yona et al., 2025).

Some microorganisms delivered by mud rain provide potential benefits for soils in terrestrial ecosystems. Among the genera (Figure 8B), *Sphingomonas* and *Pseudomonas* may actively participate in nitrogen (N_2_ fixation, denitrification) (Lowman et al., 2016; Li et al., 2022b; Ramond et al., 2022) and phosphorus (phosphate solubilization) cycles (Plante, 2006; Mulissa et al., 2016), and certain species might also be able to degrade organic compounds such as PAHs (Chen et al., 2008; Karishma et al., 2015). Other genera such as *Devosia* and *Ochrobactrum* may play a crucial role in bioremediation of metal-contaminated soils, due to their high tolerance to heavy metal concentrations according to previous studies (Pandey et al., 2013; Talwar et al., 2020).

Muddy rain may also act as a vehicle for plant-beneficial bacteria (Figure 8B). *Brevundimonas, Variovorax, Methylobacterium*, and *Flavobacterium* may include species with plant growth-promoting (PGP) traits according to literature (Soltani et al., 2010; Yin et al., 2021; Zhang et al., 2021; Zaim and Bekkar, 2023; Haedar et al., 2024; Yurimoto, 2025). Other genera such as *Rhizobium* and *Mesorhizobium* might also be able to establish mutualistic symbioses with plants based on previous studies (Dludlu et al., 2018; Verma et al., 2020).

Airborne bacteria deposited through muddy rain may also exert negative effects on ecosystem health. Certain genera, such as *Pseudomonas, Acinetobacter*, and *Sphingomonas*, may include opportunistic pathogens associated with infections in humans, plants, and animals (Stern et al., 2021; Iakovides et al., 2022; Erkorkmaz et al., 2023; Reynaud et al., 2024; Sati et al., 2025; Sun et al., 2026). In addition, some strains belonging to *Stenotrophomonas* and *Ochrobactrum* are also regarded as potential pathogens (Das et al., 2023).

### 4.4 Identification of isolated cultivable microorganisms

A total of thirty-four cultivable microorganisms (nmc) were isolated from muddy rain samples (Figure 9). The mean value was around eight isolates per sample, with a variable range from six to fourteen. Additionally, four microorganisms were also isolated from the rainwater control (RC) sample.

The highest number of isolates corresponded to LLB16, collected during a very intense African dust event (DD = 16.4 g/m^2^; PM_10_ = 550 μg/m3) (Table 1), as mentioned before. Previous studies also reported a major microbial load during very intense dust events originating from North Africa and Middle East deserts (Goudarzi et al., 2014; Amarloei et al., 2020; Chatoutsidou et al., 2023).

Most isolates were bacilli (88%), including Gram-positive (47%) and Gram-negative (41%); the remaining were Gram-positive cocci (11%) (Figure 9). Our results were consistent with previous studies on muddy rain (Navarro et al., 2024a). Generally, Gram-negative bacteria tend to be predominant in rainwater and other atmospheric waters (Šantl-Temkiv et al., 2015; Amato et al., 2017; Hu et al., 2018), whereas Gram-positive bacteria are more abundant in air and atmospheric dust (Sánchez De La Campa et al., 2013; Goudarzi et al., 2014; Els et al., 2019). According to (Itani and Smith 2016), muddy rain represents a special case in which microbial populations from both atmospheric dust and rainwater coexist and are co-transported.

Fourteen cultivable microorganisms were taxonomically identified (Table 4); an isolate from the rainwater control (RC) sample was also identified. Most of them belonged to the phyla *Pseudomonadota* (64%) and *Bacillota* (21%), with minor representation of *Actinomycetota* and *Bacteroidota* (both 7%) (Figure 10; Table 4). Our results coincided with the main phyla found in other studies on muddy rain (Navarro et al., 2024a) and atmospheric dust (Yan et al., 2022; Navarro et al., 2024b). On the other hand, the number of phyla identified through cultivation-based analyses is considerably lower than that detected using metagenomic approaches. According to literature, culture media and incubation conditions strongly bias the culturable microbial fraction, which in certain environments accounts for less than 1% of the total microbial population (Riesenfeld et al., 2004; Sharon and Banfield, 2013).

At the species level (Table 4), most isolates had been previously reported in soils, such as *Pseudomonas shirazensis* and *Peribacillus frigoritolerans* (Girard et al., 2021; Montecillo and Bae, 2022). Interestingly, other species such as *Pseudomonas poae* were associated with the vegetation and the phyllosphere in particular according to previous studies (Behrendt et al., 2003).

The case of *Peribacillus frigoritolerans* (formerly *Brevibacterium frigoritolerans*) is particularly noteworthy, as it is an extremophilic microorganism (Zhang et al., 2019) first isolated from arid soils in Morocco (Delaporte and Sasson, 1967). Additionally, this species has been recently detected in other studies on muddy rain and atmospheric dust from North Africa (Navarro et al., 2024a,b).

Among the identified isolates (Table 4), several species potentially exerting beneficial effects at the ecosystem level were also found. *Stenotrophomonas rhizophila* and *Acidovorax radicis* may act as plant growth promoters according to literature (Li et al., 2012; Elhosieny et al., 2023). Other species such as *Bacillus siamensis* may contribute to soil bioremediation as stated by (Awan et al. 2020). On the other hand, some strains of *Brevundimonas bullata* have been proposed as biocontrol agents due to their nematocidal activity in other studies (Li et al., 2022a).

## 5 Conclusions

This study provides new insights into the complex interplay between African dust transport events and the deposition of microorganisms through muddy rain in the city of Granada (Spain). By means of metagenomic analysis, electron microscopy, and the characterization of cultivable microorganisms, we demonstrate that muddy rains act as an efficient vector for the transport and dispersal of diverse and functionally significant bacterial communities.

The physical properties -mainly composed of clay-sized particles- and the mineralogical composition -dominated by phyllosilicates and quartz- of particulate matter in muddy rain may be potentially favorable for microbial growth. Additionally, microbes are frequently associated with clay particles which might offer multiple attachment surfaces and protective shelters against harsh environmental conditions.

Microscopic observations further revealed bacteria adhering to mineral particles, suggesting possible active microbe-mineral interactions and the potential formation of stable microaggregates. These findings suggest that mineral particles are not merely passive carriers but rather active components in complex biogeochemical networks where microorganisms and minerals coexist and potentially cooperate.

Muddy rain is not solely a meteorological or mineralogical phenomenon but also a biologically and ecologically relevant process of global importance, linking distant ecosystems through the exchange of living and mineral matter. Further investigation is needed for an in-depth analysis on the ecological implications of airborne microbes and their roles in global microbial dynamics, as well as in ecosystem responses to climate change and advancing desertification.

## Data Availability

The data presented in the study are deposited in the European Nucleotide Archive (ENA) repository, accession number PRJEB108927.
